# Single Voxel Proton Spectroscopy for Neurofeedback at 7 Tesla

**DOI:** 10.3390/ma4091548

**Published:** 2011-09-15

**Authors:** Yury Koush, Mark A. Elliott, Klaus Mathiak

**Affiliations:** 1 Department of Psychiatry, Psychotherapy and Psychosomatics, RWTH Aachen University, Pauwelsstrasse 30, 52074 Aachen, Germany; E-Mail: kmathiak@ukaachen.de; 2 JARA, Translational Brain Medicine, 52074 Aachen, Germany; 3 Institute of Neuroscience and Medicine (INM-1), Research Center Jülich, 52425 Jülich, Germany; 4 Center for Magnetic Resonance and Optical Imaging (CMROI), Department of Radiology, University of Pennsylvania, PA 19104, USA; E-Mail: melliott@mail.med.upenn.edu

**Keywords:** neurofeedback, signal processing, spectroscopy, imaging

## Abstract

Echo-planar imaging (EPI) in fMRI is regularly used to reveal BOLD activation in presubscribed regions of interest (ROI). The response is mediated by relative changes in T2* which appear as changes in the image pixel intensities. We have proposed an application of functional single-voxel proton spectroscopy (fSVPS) for real-time studies at ultra-high MR field which can be comparable to the EPI BOLD fMRI technique. A spin-echo SVPS protocol without water suppression was acquired with 310 repetitions on a 7T Siemens MR scanner (TE/TR = 20/1000 ms, flip angle α = 90°, voxel size 10 × 10 × 10 mm^3^). Transmitter reference voltage was optimized for the voxel location. Spectral processing of the water signal free induction decay (FID) using log-linear regression was used to estimate the T2* change between rest and activation of a functional task. The FID spectrum was filtered with a Gaussian window around the water peak, and log-linear regression was optimized for the particular ROI by adoption of the linearization length. The spectroscopic voxel was positioned on an ROI defined from a real-time fMRI EPI BOLD localizer. Additional online signal processing algorithms performed signal drift removal (exponential moving average), despiking and low-pass filtering (modified Kalman filter) and, finally, the dynamic feedback signal normalization. Two functional tasks were used to estimate the sensitivity of the SVPS method compared to BOLD signal changes, namely the primary motor cortex (PMC, left hand finger tapping) and visual cortex (VC, blinking checkerboard). Four healthy volunteers performed these tasks and an additional session using real-time signal feedback modulating their activation level of the PMC. Results show that single voxel spectroscopy is able to provide a good and reliable estimation of the ΒΟLD signal changes. Small data size and FID signal processing instead of processing entire brain volumes as well as more information revealed from the acquired total water spectrum, *i.e.*, direct estimation of the T2* values and B0 changes, make SVPS proton spectroscopy suitable and advantageous for real-time neurofeedback studies. Particular challenges of ultra-high field spectroscopy due to the non-linearity in the spectral information, e.g., poor main magnetic field homogeneity and the absence of motion correction for the SVPS sequence may lead to the special artifacts in the control signal which still need to be addressed. The contrast to noise ratio (CNR), experimental statistic (t-values) and percent signal change were used as quality parameters to estimate the method performance. The potential and challenges of the spectroscopic approach for fMRI studies needs to be further investigated.

## 1. Introduction

Real-time fMRI based on the EPI technique is frequently used for neurofeedback applications based on BOLD activation. A fMRI Brain Computer Interface (BCI) enables subjects to observe the BOLD signal in specific brain regions in real-time with a typical delay of less than 2 s from the acquisition of each echo-planar image [1,2]. In neurofeedback applications, subjects learn to control the activation level in a specified region of interest (ROI) of their brain in this manner, voluntary control of psychological functions such as mood, memory, or attention can be obtained [3,4,5,6,7,8,9,10,11,12,13]. The underlying BOLD signal in EPI is mediated by relative changes in T2* which appear as temporal changes in the image pixel intensities. The feedback signal is typically the result of averaging across correspondent image pixels of a ROI converted to a subject interface (e.g., visual projection). The fMRI approach provides thorough imaging information and allows several corrections in real time, e.g., motion correction. During the recent decade the interest in real-time fMRI has significantly grown [3,4,5,6,7,8,9,10,11,12,13]. Neurofeedback fMRI has several advantages in contrast to earlier neurofeedback methods (e.g., EEG [14,15]), including higher spatial resolution, superior localization, and increased sensitivity.

Several attempts have been made to develop an alternative approach to improve the sensitivity and specificity to the BOLD signal changes as well as provide a new functional mechanisms for revealing brain activation more precisely and effectively. One such alternative is functional near infrared spectroscopy (fNIRS; [16,17,18,19]), which measures the concentration changes of hemoglobin in response the neural activity. fNIRS is more tolerant to motion, has significantly lower costs and is portable and quiet in comparison to the fMRI approach. The main advantages of fNIRS over fMRI is its high temporal resolution (10Hz). The disadvantage however is much lower spatial resolution (3 cm) [19].

Functional single voxel spectroscopy (SVPS) has also been utilized to measure changes in T_2_* from the unsuppressed water spectrum [20]. Its feasibility has been shown for long and short TR and TE at relatively large voxel sizes (2 × 2 × 2 cm^3^ at 2T). The positive BOLD effect was also shown to be reduced with increasing TE [21].

Richards [22] suggested that functional spectroscopy can be a more direct measure of cellular activation than the fMRI technique, and discussed the spectroscopy-detectable metabolites. Mulkern [23] showed that fast magnetic resonance spectroscopic imaging (MRSI) of water based on either gradient or spin echo trains is feasible for identification of BOLD mechanisms and rapid mapping of proton changes from brain metabolites, e.g., lactate, creatine, GABA. The authors also stated that spectroscopic methods targeting ^31^P signals from phosphocreatine (PCr) and adenosine tri-phosphates (ATP) are also technically possible and may reveal cerebral energetic within fMRI contexts with higher field strength.

During the last decades several fast MRI techniques were proposed to obtain magnetic resonance spectroscopic imaging (MRSI) information [24,25,26,27,28,29]. The sensitivity-encoded (SENSE) MRSI phantom-replacement technique was able to provide reasonable N-acetyl aspartate (NAA), Choline and Creatine concentrations [30]. Long acquisition time (about tens of seconds) for MRSI [25,26,30] unfortunately cannot be appropriate for real-time studies where 1–2 sec feedback is required.

It has also been shown that metabolite ratios (Choline/NAA) obtained with SVPS spectroscopy and MRSI by point-resolved spectroscopic (PRESS) technique [31] were correlated, however, they were higher for the SVPS technique. Operating at high and ultra-high field strength increases the spectral resolution and sensitivity to the spectral changes [32,33,34]. However, the metabolite signals have very low sensitivities and rapid acquisition (*i.e.*, single average) remains challenging for real-time feedback purposes.

Single-voxel proton spectroscopy has been used to investigate the metabolism of the activated visual cortex at 7 Tesla [35]. Visual cortex was localized with help of the fMRI EPI localizer (checker board). The water signal was suppressed to reveal the metabolism. Concentration changes of greater than 0.2 μmol/g were detected in subjects for most metabolites.

It remains a challenge to achieve a sufficient quality of the feedback data which suffer from poor signal-to-noise ratio [36,37]. Contingent neurofeedback requires online detection of activity in a single trial, which enables subjects to learn the localized regulation of the own brain activity. Ultra-high field (≥7 Tesla) may enable neurofeedback of structures which are not accessible to systems with lower BOLD sensitivity and CNR. At 7 Tesla, high SNR in EPI [38,39], and high sensitivity to the SVPS acquisition [35] are well established, but feasibility as BCI still needs to be verified. The fMRI data quality remains particularly important [40,41] and therefore the additional online signal processing developed in our previous research was used [42].

In this study, we have developed an approach which provides a new look into the neurofeedback mechanism and processes within an activated ROI by measuring the characteristics of the water spectrum in real-time. The functional single-voxel proton spectroscopy (fSVPS) method is proposed and its several potential advantages are noted. The proposed method performance was assessed with the standard quality measures: CNR, experimental statistic and percent signal change. The online signal processing approach was applied in real-time neurofeedback experiments to remove the low-frequency feedback signal drift with an exponential moving average (EMA) algorithm [19,43] and the high-frequency noise and large signal outliers with a modified Kalman filter [42]. The processed feedback signal was normalized to a relative displayed range. The particularly specific demands were emphasized to develop a reliable feedback framework on an ultra-high magnetic field (7T) MR scanner.

## 2. Methods Section

### 2.1. Spectral Processing of the Water Signal

Functional single voxel proton spectroscopy (fSVPS) estimates the functional changes in T_2_* from the unsuppressed water spectrum. The raw FID signal measured in the time domain underwent the following processing steps in order to extract the control signal: central frequency shift, filtering in frequency domain with Gaussian window, log-linear regression in the time domain, and regression optimization. All operations were performed online for neurofeedback studies except linearization optimization, which was applied before the neurofeedback session to achieve an individual optimization of performance. The optimization procedure was based on data acquired from an fSVPS finger tapping paradigm without feedback.

**Figure 1:**
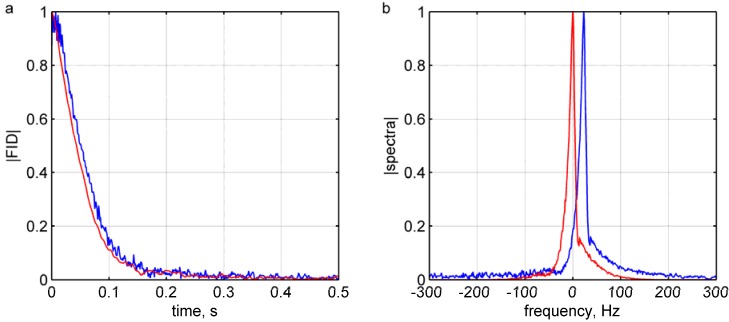
Spectral processing of the single voxel spectroscopic data. (**a**) Raw (blue) and processed (red) magnitude free induction decay functions (FIDs); (**b**) Raw (blue) and processed (red) magnitude spectra. Note that graphics and spectra were normalized to their maximum values for comparison purposes and the panels are scaled differently.

The free induction decay function (FID) in the time domain can be described in complex form as a function of time with a set of exponential components (Figure 1a):

(1) $$FID(t)=\sum_{j=1}^{N}A_{j}e^{i2\pi f_{j}t}e^{i\phi_{j}}e^{-t/T_{2j}^{*}}$$

with amplitudes *A_j_*, frequencies *f_j_*, relaxation times T_2,j_* and constant phases *Φ_j_*.

The detection of the BOLD effect focuses on the main water component in the signal by its apparent change in T2* due to fluctuations in the microscopic field homogeneity. The water signal consists of several components, including both intravascular and extravascular compartments of gray and white matter, as well as cerebro-spinal fluid (CSF). As with the BOLD signal from EPI, the T2* changes in the CSF are negligible and produce only a constant contribution to the overall T2* value and an unsystematic noise component. After the central frequency shift and Gaussian filtration (frequency domain), the off-resonance signal contaminations from lipids and metabolites are removed and the FID is approximated by a single water component:

(2) $$F\hat{I}D(t)=A_{water}e^{i2\pi f_{water}t}e^{i\phi_{water}}e^{-t/T_{2water}^{*}}$$

and for *f_water_ ≈* 0 after the central frequency shift Equation 2 can be simplified to:

(3) $$F\hat{I}D(t)=A_{water}e^{i\phi_{water}}e^{-t/T_{2water}^{*}}$$

**Figure 2:**
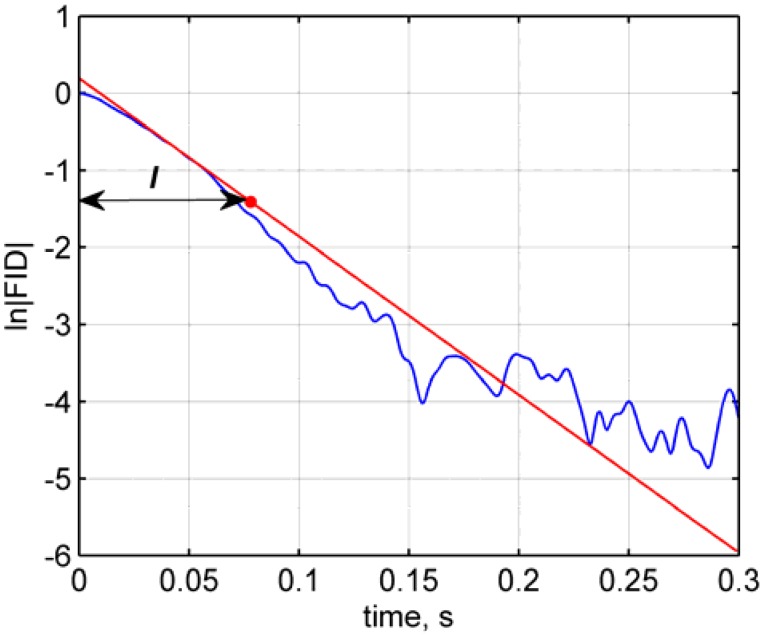
Linear regression of FID data. The linear regression fit (red) is exemplarily shown for a VC fSVPS single voxel *ln(FID)* data (blue) and its optimal linearization length (red dot; *l_optim_ =* 0.078 s, *t* = 19.12, p < 0.001).

Taking the logarithm (*ln*) of (3) we get:

(4) $$\text{ln}(FID)=-t/T_{2water}^{*}+\text{ln}(A_{water})+i\phi_{water}$$

which is the equation of a complex straight line. Therefore, applying complex domain linear regression to the logarithm of the measured FID series we can estimate the T_2_* of the joint water signal change between rest and activation of a functional task (Figure 2). However, the processed signal in Equation [4] may still have a large non-linear portion due to poor shim and residual contamination from other signals (e.g., lipids) (Figure 2; blue curve). This condition complicates the straight-forward regression analysis of the acquired data. The linear regression length (*l*) can be optimized in the sense of a statistical measure (*i.e.*, time series t-value as a function of *l*). On the other hand, taking the whole data length for linear regression can produce a large and non-systematic error in T_2_* estimation. The linearization length function can be very specific in a particular ROI.

### 2.2. Real-Time Feedback Signal Processing

The online feedback signal processing includes four consecutive operations: (1) signal drift removal with exponential moving average (EMA); (2) spike detection and correction; (3) high frequency noise removal; and (4) feedback signal normalization. Operations 2 and 3 are composite and performed within one estimator, *i.e.*, the modified Kalman filter. The signal processing methods were configured once before all experiments were conducted (for details and validation see [42]).

#### 2.2.1. EMA as a High-Pass Filter

Feedback signal drift was removed with the exponential moving average (EMA) algorithm which was approved as an effective tool for neuroimaging applications (e.g., in fNIRS, see Cui [19]). Performance of the EMA filter depends on the value of the smoothing factor (α). The cut-off frequency of the applied filter was 0.003 Hz (α = 0.98, repetition time TR = 1 sec, filter time constant τ = 49) and thus well-below the required BOLD frequency range (0.01–0.12 Hz; [44,45,46]).

#### 2.2.2. Non-Linear Modified Kalman Filter as Low-Pass Filter and Spike Detector

In its linear form, a Kalman filter is an adaptive estimation (filtering) algorithm extracting the desired signal from the observation input [47,48,49]. It therefore can be used as a low-pass filter in signal processing applications. By non-linear modification, the Kalman filter can be adapted for spike detection and rejection [42]. The inequality

(5) $$\left|K_{m}(y_{m}-H\cdot x_{m}^{-})\right|>Th_{sp}$$

with the threshold *Th_sp_* and update term *K_m_(y_m_ – H·x^–^_m_)* was used to detect the unexpected Kalman estimator updates (spikes). The threshold

(6) $$Th_{sp}=0.9\cdot std(y_{0}...y_{t})$$

with the incrementally calculated standard deviation *std(y_0_…y_t_)* of the input raw time series up to the given time point *t* was used in the presented neurofeedback studies. The non-linear spike removal influences the spectral properties of the filter and the estimated covariance. Kalman filter update ratio λ = 4 was selected to keep the cutoff frequency around 0.1 Hz (≈π^−1^ λ^−1^).

#### 2.2.3. Normalization of the Feedback Signal

The feedback signal normalization was used at a final signal processing step to adopt the signal to the display range. Incrementally updated local signal maximum and minimum were used to assign the normalization range based on the 1% expected BOLD effect threshold. Once the normalization range reached its maximum it was not reduced before the end of the session thus assuming that if a subject reached certain activation level maximum, he could keep on this level or even do better. However, different normalization strategies could be used (compare [1,6,50]).

The spectral and feedback signal processing modules are the parts of an in-house developed Matlab 7.9 (The Mathworks, Natick, MA, USA). The visualization module was incorporated into the neurofeedback toolbox based on Cogent routines (http://www.vislab.ucl.ac.uk/cogent.php, 2009). Subjects viewed the display projected on the MR-compatible screen. The software is available on request from the corresponding author.

### 2.3. Method Performance Assessment Parameters

The contrast to noise ratio, experimental statistic and percent of signal changes were used to quantify the proposed method performance. All quality measures were estimated for the stored feedback signal after all appropriate preprocessing and the feedback signal processing operations were applied except normalization. The contrast to noise ratio (CNR) was used to estimate the sensitivity to the BOLD signal changes:

(7) $$CNR=\frac{mean(cond)-mean(bas)}{\sqrt{\text{var}(cond)+\text{var}(bas)}}$$

with the block-wise averages *mean(cond)* and *mean(bas)* during the baseline and condition blocks respectively; *var(cond)* and *var(bas)* being the corresponding variances.

Statistical analysis of the acquired datasets was used to investigate the significance of fMRI signal changes in each session with the experimental block-design model prepared in SPM8 (Welcome Trust Center, UK). Subjects’ motion parameters were not included into the GLM for spectroscopic data statistical analysis as single voxel spectroscopy sequence doesn’t have the motion correction inbuilt. In contrast, for EPI sequences the motion parameters were included. We also compared EPI statistical data analysis with and without including the motion parameters which does not make much difference for, e.g., *t-* and *p-*group average values because of the very small head motion in our study (less then ±0.5mm). Notably, the selected ROI was smaller than whole activated region of the PMC or VC area. Even within potentially relatively large head movements (e.g., ±3 mm) the presubscribed ROI would remain within the activated regions at all time points. We neglected the motion correction for our spectroscopic data statistic due to technical limitations. In the future, motion compensation would be advisable. For instance, navigator echoes or video monitoring could be used for a prospective motion correction by moving the voxel with the subject’s head.

Percent of signal change (*percent*) is a quality measure which can be calculated without scaling in an event-wise manner:

(8) $$perc=\frac{mean(cond)-mean(bas)}{mean(bas)}$$

However, it does not include the noise variances in that form.

## 3. Experimental Section

### 3.1. Neurofeedback Data Acquisition and Preprocessing

The fMRI and fSVPS data were acquired on 7T MRI scanner (Siemens Medical Solutions, USA) using single-shot gradient-echo T2*-weighted EPI and spin-echo SVPS without water suppression. EPI sequence protocol acquired 300 BOLD images for PMC and VC functional localizers with TE/TR = 28/1000 ms, 16 slices volumes, matrix size 64 × 64, voxel size = 3 × 3 × 3.75 mm^3^, flip angle α = 77°, and bandwidth 2.23 kHz/pixel. A spin-echo SVPS protocol was also acquired with 300 repetitions, TE/TR = 20/1000 ms, flip angle α = 90°, average voxel size 10 × 10 × 10 mm^3^, bandwidth 1 kHz. Spectroscopic signal quality was improved by manual calibration of the transmitter amplitude and by optimizing the gradient shim currents using first the automated Siemens “advanced” auto-shimming feature, followed by manual adjustment of the shim currents. The entire calibration and shimming procedure required approximately 4 minutes. A single channel quadrature head coil received the radio-frequency MR signal.

The reconstructed images in DICOM format and single-voxel 1-dimensional FIDs data files were exported to a personal computer (PC) running with Windows XP SP2 operating system. An additional Siemens ICE functor was custom written and incorporated into the spectroscopy sequence reconstruction chain to provide the real-time export to the desired location via local network. A special graphical user interface (GUI) to interactively select the ROI on the EPI BOLD activation map and the routine to prescribe the single-voxel coordinates based on EPI coordinates were developed. Exported fMRI data were preprocessed in real-time on the local PC, *i.e.*, realigned with 3D motion correction (SPM8 routines; Welcome Trust Center, UK) to the preselected template and spatially smoothed.

### 3.2. Experimental Design

In order to investigate the sensitivity of the proposed method to the BOLD signal changes, experimental procedures targeting the primary motor cortex (PMC) and visual cortex (VC) were designed. Both ROIs show robust BOLD activity and have been previously employed in fMRI-BCI experiments. Therefore they seemed suitable to test the sensitivity of the suggested system. Moreover, VC activation was expected to be less sensitive to fatigue or variable performance (e.g., different finger tapping activity). The functional EPI localizer paradigm was applied first in the PMC and VC experiment to identify the corresponding ROIs. Subsequently, a single rectangular-shaped fSVPS voxel was located over the most active region according to the t-statistic activation map and subjects performed the same paradigm again. Finally, to test the feasibility of the proposed fSVPS approach for real-time applications such as neurofeedback, subjects performed an additional session modulating their activation level of the PMC in real-time (PMC NF). The feedback signal was extracted from the real-time (online) fSVPS data and displayed to the subject every 1 s. Systematic testing of learning of voluntary control and psychological effects of the neurofeedback were not within the scope of the present study.

Acquired data from four healthy volunteers (4 males, an average age 27) were organized in two datasets. The first dataset encompassed three subsequently performed sessions targeted PMC: the EPI localizer, the fSVPS offline session and fSVPS neurofeedback. Standard finger tapping offline and neurofeedback paradigms comprised of 5 baseline blocks and 5 activation blocks lasting 30 seconds each. Subjects were instructed to tap the non-dominant (left) arm fingers during the control blocks (TAP cue) and do not move the arm during the baseline blocks (fixation dot cue). During the neurofeedback session subjects were instructed to find an optimal individual self-control strategy to regulate the activation level of the PMC by changing the tapping strength or speed. During the neurofeedback block an additional cue (red horizontal bar) appeared in the upper part of the screen indicating the aim for the control and the position of a moving bar (green horizontal bar) encoded the activity level in the preselected ROI. The 300 volumes of subject performance for EPI functional localizer and voxels’ FIDs for fSVPS offline as well as for neurofeedback sessions were acquired with TR = 1 s; the first 10 volumes were discarded to avoid T1 saturation effect.

The second dataset comprised two subsequent sessions targeted VC: the EPI localizer and fSVPS offline session. The timing diagram used was the same as for the first dataset. Subjects were instructed to watch the screen with blinking checkerboard (6 Hz) during the activation block and fixation dot during the baseline respectively.

## 4. Results and Discussion

**Figure 3:**
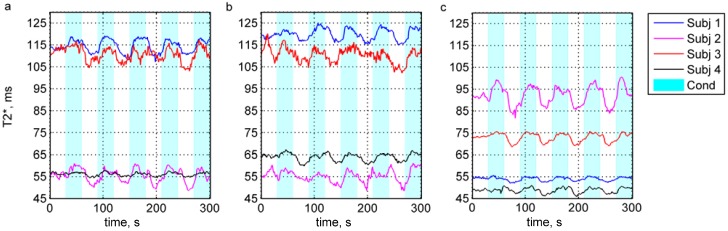
Functional single-voxel proton spectroscopy (fSVPS)-estimated T2* time series. (**a**) The primary motor cortex (PMC); (**b**) PMC in real time (PMC NF); and (**c**) Visual cortex (VC) functional single-voxel proton spectroscopy (fSVPS) time series (processed) are displayed for four control subjects. Acquired time series show significant variability between subjects and regions of interest (ROIs) and overall higher than expected T2* values.

Single voxel spectroscopy provided a measure for ΒΟLD signal changes as manifested by the spectroscopy time series for PMC, PMC NF, and VC conditions in all four investigated subjects (Figure 3). Although the T2* values show detectable dynamic change in the measured regions due to the BOLD activation, the absolute T2* values differ from those expected at 7T for venous blood (T2* < 5 ms), gray matter (T2* ~ 21 ms), and arterial blood (T2* ~ 40 ms) [51,52]. The baseline T2* values averaged across the four subjects were: PMC (T2*_offl_ = 84.2 ± 2.5), PMC NF (T2*_nf_ = 87.3 ± 2.6), and VC (T2*_offl_ = 67.0 ± 2.1). These large calculated T2* values indicate that the measured signal contains spin-echo components from the SVS excitation scheme and partial volume effects with cerebro-spinal fluid–particularly in some PMC ROIs. Thus, for the estimation of absolute T2* values, a multicomponent model could be considered instead of the simplification we introduced in Equation 2.

**Figure 4:**
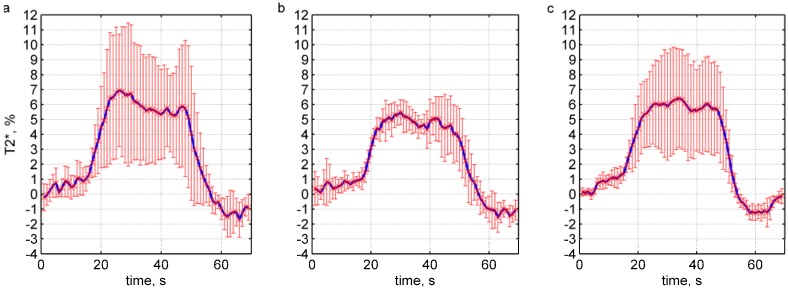
Time-locked T2* changes. Group event-related averages (blue) and their SD across subjects (red bars) for (**a**) PMC; (**b**) PMC NF; and (**c**) VC fSVPS time series are shown. Note that PMC NF time series showed much lower event-related standard deviations but also lower average signal change (b; see also Figure 5c).

Group analysis of time-locked T2* changes revealed a typical BOLD response to a block design with T2* changes of 5% or more (Figure 4). An apparent effect of the neurofeedback was a decreased standard deviation (SD) in the BOLD response (PMC: SD = 2.3 ± 1.5; PMC NF: SD = 1.0 ± 0.5; VC: SD = 1.7 ± 1.3; see Figure 4b). SNR calculation based on of the event-related blocks also yielded higher values for the NF condition (SNR_PMC NF_ = 4.9 *vs.* SNR_PMC_ = 2.6). The feedback of the independent variable of this experiment may have resulted in a more consistent subject performance.

**Figure 5:**
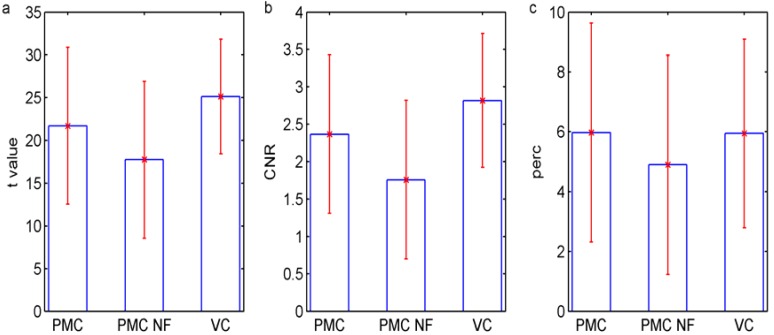
fSVPS quality measures. Average (**a**) t-value; (**b**) contrast to noise ratio(CNR); and (**c**) percent signal change (blue bars ± SD in read) show similar patterns across the PMC, PMC NF, and VC conditions. The SVPS approach provides robust statistics based on its high CNR and percent signal change. Note that the panels are scaled differently.

Additionally, contrast to noise ratios (CNRs), experimental statistics (t-values) and percent signal changes were used as quality parameters to estimate the method performances (Figure 5a-c). The t-values were calculated across the four subjects PMC (t = 21.7 ± 9.2), PMC NF (t = 17.7 ± 8.3), and VC condition (t = 25.1 ± 6.7). For the constant sample size and constant inter-subject noise, CNR and percent signal change reflected the same pattern (PMC: CNR = 2.4 ± 1.1, Δ% = 6.0 ± 3.7; PMC NF: CNR = 1.8 ± 0.8, Δ% = 4.9 ± 1.0; VC: CNR = 2.8 ± 0.9, Δ% = 5.9 ± 3.1), but also note the lower inter-subject variance in the PMC NF condition.

The applied quality measures demonstrated consistent results with highest t-values during the visual checkerboard test (VC condition). The neurofeedback sessions had the smallest average quality scores which may be explained by subject latency and adaptation to the required control task. However, the SNR of the event-related average, which was calculated across subjects, significantly benefited from the neurofeedback approach (see Figure 4a and Figure 4b). The evaluated quality parameters revealed only small differences between PMC and VC time series. We conclude that fSVPS (PMC, VC) and real-time fSVPS with BCI feedback (PMC NF) achieve consistent and repeatable BOLD measures at different ROI locations during different experimental tasks.

**Figure 6:**
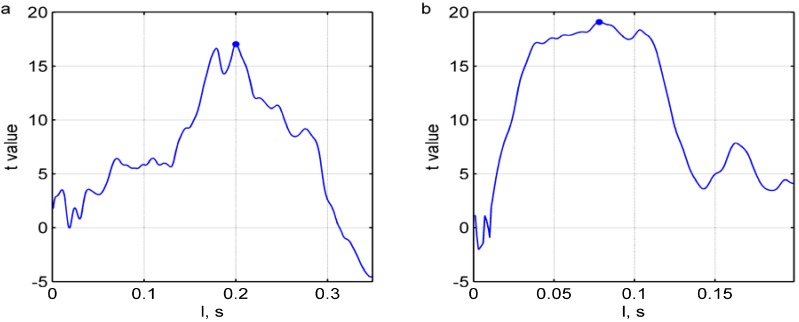
Optimal linear regression length. Experimental statistic (t-value) depends on the applied linear regression length and reveals an optimum duration, *l_optim_*, here shown in a typical example for (**a**) the PMC (blue dot; *l_optim_* *=* 200 ms, t = 17.0, p < 0.001) and (**b**) the VC condition (blue dot; *l_optim_* *=* 78 ms, t = 19.12, p < 0.001). Note that panels are scaled differently.

The sensitivity of the proposed method depends on the MR scanner field strength, its characteristics and adjustments procedures, (e.g., quality of the shimming procedure) and therefore, the spatial localization of the ROI can substantiate the stability and repeatability of the linear pattern in the *ln(FID)* of acquired spectroscopic voxels in time (Figure 6).

The maximum t-value (Figure 6) was specific for selected ROI and time from the beginning of FID acquisition. Thus, with the chosen TE = 20 ms, the maximal t-value was attained 220 ms after the first excitation pulse in the PMC example. Notably, these values depend on SNR and shim condition and varied individually. The optimum regression lengths are not directly related to BOLD or local T2* values.

Furthermore, instability or absence of plateau in the linear regression optimization lengths (Figure 5a) may result in an unexpected shift of optimal regression length (l_optim_) for the next feedback session. The loss or shift in l_optim_ can therefore result in unwanted reduction of the method sensitivity to the BOLD signal changes and requires a special attention to the ultra-high field MR scanner adjustment procedures. This finding also shows a significant spectral characteristics dependency from the spatial localization of the ROI for ultra-high MR fields.

When compared to conventional multi-slice EPI BOLD, functional spectroscopy benefits from small data size and decreased signal processing load. This low computational demand (<0.2 s) is advantageous for neurofeedback studies in which processing latency is critical. This has the potential to be exploited for ultra-fast SVPS feedback (e.g., TR < 1 sec), since the FID need only be sampled for the optimal linear regression duration (on the order of 0.2 sec). The spatial resolution in single voxel spectroscopy is comparable to single ROI based imaging approaches. Additionally, the SVPS approach averages the collective water signal more efficiently than multi-voxel imaging ROI methods, since all the desired signal is acquired in a single excitation. However, SVPS approaches are limited to rectangular prism-shaped ROIs, while BOLD imaging ROIS can contain any arbitrary pixel combination. Finally, it was observed that large voxels (greater than 2 × 2 × 2 cm^3^) in ultra-high field spectroscopy were overly compromised by poor shimming conditions.

More information revealed from the spectral characteristics, *i.e.*, direct estimation of the T_2_* values and B0 changes from the acquired total water spectrum make fSVPS more informative and sensitive to potential brain activation patterns. Particular challenges of ultra-high field spectroscopy due to the non-linearity in the spectral information, e.g., by poor main magnetic field shimming for large voxels require precise justification of the experimental set up. Motion artifacts in spectroscopic sequence were neglected in our study; however, it can be absolutely required in clinical applications with expected larger patients’ motion. The special navigator can be developed to track the head motion in between the spectroscopic voxels’ acquisitions [53,54].

## 5. Conclusions

The functional real-time single-voxel proton spectroscopy (fSVPS) method directly estimating the T_2_* values from the unsuppressed water spectrum was presented. Ultra-high static field (≥ 7 T) are suggested to achieve the required single trial sensitivity for neurofeedback. This novel approach has shown significant and reliable estimation of the BOLD signal changes for PMC and VC studies. The potential and challenges of the spectroscopic approach for functional studies on ultra-high field MR need to be further investigated.
