# Correction: Koush, Y.; Elliott, M.A. and Mathiak, K. Single Voxel Proton Spectroscopy for Neurofeedback at 7 Tesla. *Materials* 2011, *4*, 1548–1563

**DOI:** 10.3390/ma4112057

**Published:** 2011-11-24

**Authors:** Yury Koush, Mark A. Elliott, Klaus Mathiak

**Affiliations:** 1 Department of Psychiatry, Psychotherapy and Psychosomatics, RWTH Aachen University, Pauwelsstrasse 30, Aachen 52074, Germany; E-Mail: kmathiak@ukaachen.de; 2 JARA, Translational Brain Medicine, Aachen 52074, Germany; 3 Center for Magnetic Resonance and Optical Imaging (CMROI), Department of Radiology, University of Pennsylvania, Philadelphia, PA 19104, USA; E-Mail: melliott@mail.med.upenn.edu; 4 Institute of Neuroscience and Medicine (INM-1), Research Center Jülich, Jülich 52425, Germany

In the published manuscript “Koush, Y.; Elliott, M.A. and Mathiak, K. Single Voxel Proton Spectroscopy for Neurofeedback at 7 Tesla. *Materials* **2011**, *4*, 1548-1563”, all estimates of T2* from the single voxel spectroscopy data were overestimated by a factor of 4. This was due to an incorrectly assumed four-fold lower sampling rate. The focus of the manuscript is on the relative changes in T2* with BOLD activation, and not on the absolute values. Therefore, none of the central claims are affected, but the scaling in most of the figures needs to be adjusted. The authors would like to make the following corrections to their published paper.

**1.**  Figure 1, p. 1551 should be with the correct time and frequency axes:

**Figure 1:**
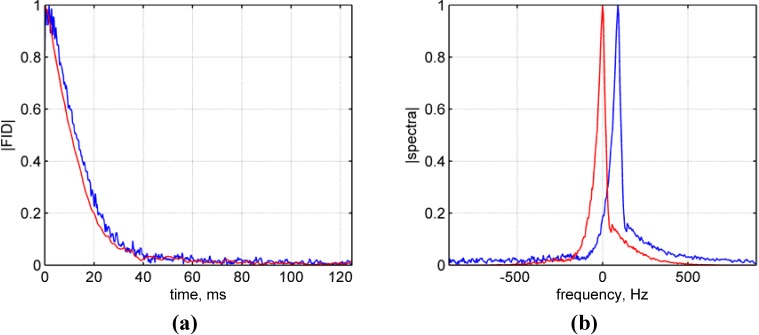
Spectral processing of the single voxel spectroscopic data. (**a**) Raw (blue) and processed (red) magnitude free induction decay functions (FIDs); (**b**) Raw (blue) and processed (red) magnitude spectra. Note that graphics and spectra were normalized to their maximum values for comparison purposes and the panels are scaled differently.
**2.**  Figure 2, p. 1552 should be with the correct time axis. The *l_optim_* should be 19.7 ms; not 0.078 s.
  **Figure 2:**
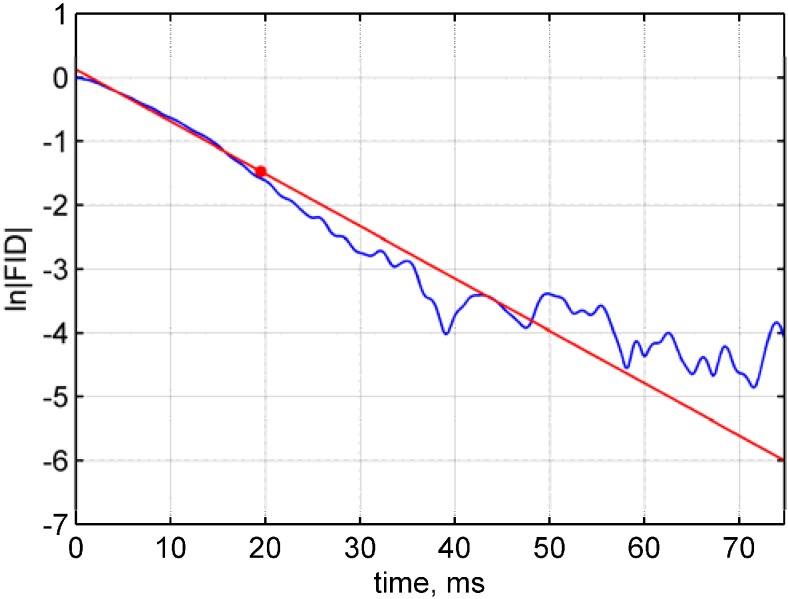
Linear regression of FID data. The linear regression fit (red) is exemplarily shown for a VC fSVPS single voxel *ln(FID)* data (blue) and its optimal linearization length (red dot; *l_optim_ =* 19.7 ms, *t* = 19.12, p < 0.001).
**3.**  Last paragraph, lines 5–7, p. 1554: For the following sentence the bandwidth should be 2 kHz; not 1 kHz. The acquisition duration = 256 ms should be specified.
  A spin-echo SVPS protocol was also acquired with 300 repetitions, TE/TR = 20/1000 ms, flip angle α = 90°, average voxel size 10 × 10 × 10 mm^3^, bandwidth = 2 kHz, acquisition duration = 256 ms.
**4.**  Second paragraph, lines 3–5, p. 1556: The sentence with citations [51,52] should be modified to “the absolute T2* values in some subjects differ from those expected”; not “the absolute T2* values differ from those expected”.
  Although the T2* values show detectable dynamic change in the measured regions due to the BOLD activation, the absolute T2* values in some subjects differ from those expected at 7T for venous blood (T2* < 5 ms), gray matter (T2* ~ 21 ms), and arterial blood (T2* ~ 40 ms) [51,52].
**5.**  Second paragraph, lines 5–7, p. 1556: The T2* estimates should be in *ms*, PMC (T2*_offl_ = 21.1 ± 0.6 ms), PMC NF (T2*_nf_ = 21.8 ± 0.7 ms), and VC (T2*_offl_ = 16.8 ± 0.5 ms); not in arbitrary units, PMC (T2*_offl_ = 84.2 ± 2.5), PMC NF (T2*_nf_ = 87.3 ± 2.6), and VC (T2*_offl_ = 67.0 ± 2.1).
  The baseline T2* values averaged across the four subjects were: PMC (T2*_offl_ = 21.1 ± 0.6 ms), PMC NF (T2*_nf_ = 21.8 ± 0.7 ms), and VC (T2*_offl_ = 16.8 ± 0.5 ms).
**6.**  Figure 3, p. 1556: The T2* axis should be modified. The last sentence of the Figure 3 description should be “Acquired time series show significant variability between subjects and regions of interest (ROIs) T2* values.”; not the “Acquired time series show significant variability between subjects and regions of interest (ROIs) and overall higher than expected T2* values.”. The sentence of the Figure 3 description ‘These large calculated T2* values indicate that the measured signal contains spin-echo components from the SVS excitation scheme and partial volume effects with cerebro-spinal fluid–particularly in some PMC ROIs.’ should be removed.
  **Figure 3:**
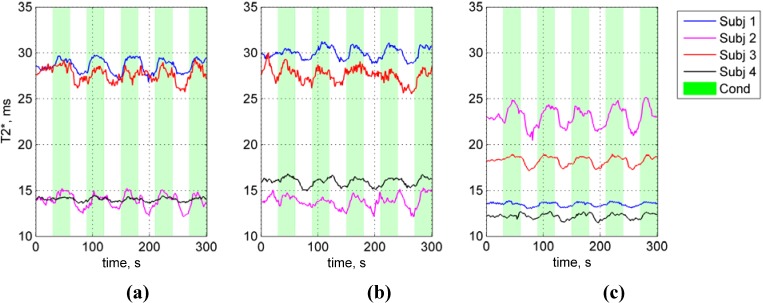
Functional single-voxel proton spectroscopy (fSVPS)-estimated T2* time series. (**a**) The primary motor cortex (PMC); (**b**) PMC in real time (PMC NF); and (**c**) Visual cortex (VC) functional single-voxel proton spectroscopy (fSVPS) time series (processed) are displayed for four control subjects. Acquired time series show significant variability between subjects and regions of interest (ROIs) T2* values.
**7.**  Figure 6, p. 1558 should be with the correct time axes. The PMC *l_optim_* should be 50.2 ms; not 200 ms. The VC *l_optim_* should be 19.7 ms; not 78 ms:

**Figure 6:**
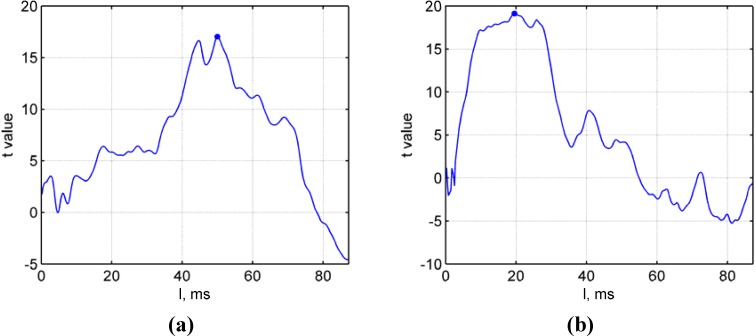
Optimal linear regression length. Experimental statistic (t-value) depends on the applied linear regression length and reveals an optimum duration, *l_optim_*, here shown in a typical example for (**a**) the PMC (blue dot; *l_optim_ =* 50.2 ms, t = 17.0, p < 0.001) and (**b**) the VC condition (blue dot; *l_optim_ =* 19.7 ms, t = 19.12, p < 0.001). Note that panels are scaled differently.
**8.**  Second paragraph, lines 2–3, p. 1558. The maximum t-value attained should be 70 ms; not 220 ms.
  The maximum t-value (Figure 6) was specific for selected ROI and time from the beginning of FID acquisition. Thus, with the chosen TE = 20 ms, the maximal t-value attained was around 70 ms after the first excitation pulse in the PMC example.

We apologize for any inconvenience caused to the readers.

